# Homeostasis of the gut barrier and potential biomarkers

**DOI:** 10.1152/ajpgi.00048.2015

**Published:** 2016-12-01

**Authors:** Jerry M. Wells, Robert J. Brummer, Muriel Derrien, Thomas T. MacDonald, Freddy Troost, Patrice D. Cani, Vassilia Theodorou, Jan Dekker, Agnes Méheust, Willem M. de Vos, Annick Mercenier, Arjen Nauta, Clara L. Garcia-Rodenas

**Affiliations:** ^1^Host-Microbe Interactomics, Animal Sciences, Wageningen University, Wageningen, The Netherlands;; ^2^Nutrition-Gut-Brain Interactions Research Centre, School of Medicine and Health, Örebro University, Örebro, Sweden;; ^3^Centre Daniel Carasso, Danone Research, Palaiseau, France;; ^4^Blizard Institute, Barts and The London School of Medicine and Dentistry, Queen Mary University of London, Whitechapel, London, United Kingdom;; ^5^Division of Gastroenterology-Hepatology, Department of Internal Medicine, University Hospital Maastricht, Maastricht University Medical Centre, Maastricht, The Netherlands;; ^6^Louvain Drug Research Institute, WELBIO (Walloon Excellence in Life Sciences and BIOtechnology), Metabolism and Nutrition Research Group, Université Catholique de Louvain, Brussels, Belgium;; ^7^Neuro-Gastroenterology and Nutrition Group, Institut National de la Recherche Agronomique, Toulouse, France;; ^8^Danone, Paris, France;; ^9^Laboratory of Microbiology, Wageningen UR, Wageningen, The Netherlands;; ^10^Institute of Nutritional Science, Nestlé Research Center, Lausanne, Switzerland; and; ^11^FrieslandCampina, Amersfoort, The Netherlands

**Keywords:** gut barrier, antimicrobial peptides, microbiota, epithelial permeability

## Abstract

The gut barrier plays a crucial role by spatially compartmentalizing bacteria to the lumen through the production of secreted mucus and is fortified by the production of secretory IgA (sIgA) and antimicrobial peptides and proteins. With the exception of sIgA, expression of these protective barrier factors is largely controlled by innate immune recognition of microbial molecular ligands. Several specialized adaptations and checkpoints are operating in the mucosa to scale the immune response according to the threat and prevent overreaction to the trillions of symbionts inhabiting the human intestine. A healthy microbiota plays a key role influencing epithelial barrier functions through the production of short-chain fatty acids (SCFAs) and interactions with innate pattern recognition receptors in the mucosa, driving the steady-state expression of mucus and antimicrobial factors. However, perturbation of gut barrier homeostasis can lead to increased inflammatory signaling, increased epithelial permeability, and dysbiosis of the microbiota, which are recognized to play a role in the pathophysiology of a variety of gastrointestinal disorders. Additionally, gut-brain signaling may be affected by prolonged mucosal immune activation, leading to increased afferent sensory signaling and abdominal symptoms. In turn, neuronal mechanisms can affect the intestinal barrier partly by activation of the hypothalamus-pituitary-adrenal axis and both mast cell-dependent and mast cell-independent mechanisms. The modulation of gut barrier function through nutritional interventions, including strategies to manipulate the microbiota, is considered a relevant target for novel therapeutic and preventive treatments against a range of diseases. Several biomarkers have been used to measure gut permeability and loss of barrier integrity in intestinal diseases, but there remains a need to explore their use in assessing the effect of nutritional factors on gut barrier function. Future studies should aim to establish normal ranges of available biomarkers and their predictive value for gut health in human cohorts.

in multicellular animals the intestinal tract is a dominant arena for interaction with commensal microbiota. The coevolution of mammals with intestinal bacteria has resulted in a highly specialized mucosa that can fulfill the requirement for digestion and absorption of nutrients while maintaining a peaceful coexistence with symbionts and protecting the body against infection. In this respect the chemical and physical components of the intestinal mucosa, often referred to as the “gut barrier,” play a crucial role. A consequence of perturbations in gut barrier function, for example due to poor nutrition, infection, or other illness, can lead to increased “intestinal permeability,” which refers to the rate of flux of molecules across the epithelium. Thus, the terms “gut barrier” and “intestinal permeability” are often used interchangeably, although they refer to different functional aspects of the mucosa. Increased intestinal epithelial permeability, also known as “leaky gut,” is associated with a variety of gastrointestinal disorders, including inflammatory bowel disease (IBD), irritable bowel syndrome (IBS), celiac disease, and the early stages of colon cancer development (27). In IBD, altered permeability increases the translocation of proinflammatory stimuli into the lamina propria (LP), triggering inflammatory cytokine-mediated changes to the tight junctions that result in permeability changes (46). Similarly, the increased epithelial permeability associated with the diarrheal form of IBS is thought to exacerbate the symptoms via increased paracellular transport of luminal antigens (20). Altered intestinal epithelial permeability is furthermore associated with type 2 and type 1 diabetes, celiac disease, and food allergy among others (62, 76, 199, 203, 278). Consequently, modulation of gut barrier function is a highly relevant target for novel treatment and prevention strategies against a range of diseases that have all increased dramatically over the past 5 decades. Nutrition and microbe-gut interactions can have a substantial and clinically relevant effect on the development of the immune system and intestinal barrier function with consequences for resistance to pathogens, development of gut inflammation, and abdominal complaints (131).

In this review, we describe the role of different defense mechanisms that support barrier function, and how they are regulated and measured. Additionally, we describe how integrity of the barrier is maintained and regulated by the complex network of interactions between microbes and host epithelium. Finally, we discuss the importance of proper functioning of the gut barrier in relation to bidirectional signaling between the enteric nervous system and the brain. We end the review by discussing biomarkers in blood, feces, or urine that can be used to assess intestinal permeability and epithelial integrity as well as ex vivo approaches to studying gut barrier function and intestinal permeability.

## Intestinal Epithelium, Tight Junctions, and Gut Permeability

A single-cell epithelial layer separates the intestinal luminal content from the underlying loose connective tissue and the interior milieu. The intestinal epithelium is renewed every 3–5 days in humans due to apoptosis and exfoliation of mature enterocytes and their replacement by proliferation from stem cells in the crypts. The high rate of epithelial cell turnover also serves as a protective mechanism to remove infected or damaged cells (27, 91). Increased epithelial cell proliferation will increase cell crowding at the villus tip, which is known to be a driver of epithelial cell extrusion (64). Stem cells found in the crypts of the small intestine and colon have the ability to perpetuate themselves and the potential to generate differentiated cells of the tissue of origin, a process otherwise known as multipotency (47). Intestinal stem cells continuously generate rapidly proliferating transit-amplifying cells, which differentiate into mature enterocytes, goblet cells, or endocrine cells after migrating upward and out of the intestinal crypt (295). In the small intestine, stem cells also differentiate into Paneth cells during downward migration to the base of the crypt, where they reside below the stem cell population (230). Paneth cells help to maintain the stem cell niche through paracrine signaling and they regulate the proliferation and differentiation programs of other cell lineages (22). Paneth cells also play a key role in innate immunity discussed below in *Antimicrobial Peptides and Proteins*. In colon crypts, cells expressing CD24 that reside between the stem cells may represent may represent Paneth cell equivalents (229).

**Fig. 1.**
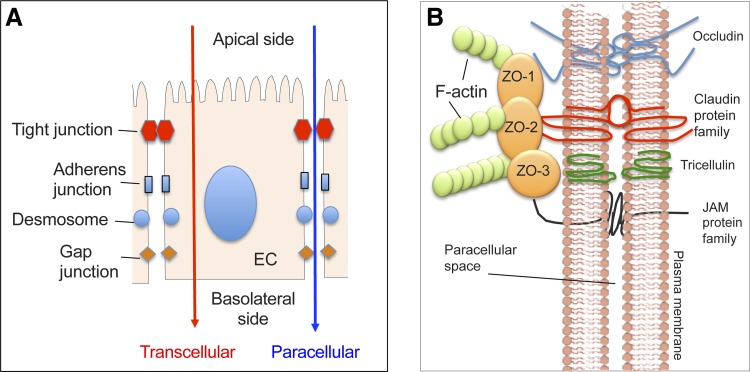
*A*: simplified schematic view of the location of the cellular junctions in juxtaposed epithelial cells (EC). Tight junctions (TJs) form the most apical junction and interconnect laterally neighboring cells in the epithelium. TJs allow selective diffusion of fluids, electrolytes, and small molecules through the paracellular space while providing a highly selective barrier for larger molecules, thereby regulating paracellular permeation of ions and other molecules. Adherens junctions are involved in cell-cell adhesion and intracellular signaling. Other basolateral epithelial junctions include desmosomes and gap junctions, which are involved in cell-cell adhesion and intracellular communication, respectively. *B*: TJs are composed of several types of occludins, junctional adhesion molecule (JAM) proteins, and members of the claudin protein family that influence the charge selectivity of the TJ. These are all transmembrane proteins that form intermolecular and intercellular connections within the paracellular space. All transmembrane junctional proteins interact with intracellular scaffold proteins (such as ZO-1, -2, and -3) that interact with other proteins, including actin in the cytoskeleton.

The paracellular permeability of the epithelium is controlled by protein complexes known as tight junctions (TJs), which reside near the apical surface of adjacent epithelial cells (Fig. 1). TJs prevent the paracellular passage of large molecules through the epithelium while allowing diffusion of ions, water, and small compounds [reviewed in (257)]. Beneath the TJs are the adherens junctions, desmosomes, and gap junctions, which are lateral structures involved in cell-cell adhesion and intracellular signaling (284). Both TJs and adherens junctions are connected to the cellular actin cytoskeleton (Fig. 1). The TJs also demarcate the apical and basolateral membranes of epithelial cells by preventing membrane diffusion of lipids, receptors, and other membrane proteins through the junction complex Fig. 1 (294). Integrins in the basolateral membrane of the epithelium are attached to the extracellular matrix present in the underlying connective tissue of the lamina propria (LP).

The permeability of the epithelium varies along the intestinal tract, and is determined by the composition and abundance of different components of the TJ. The TJ consist of transmembrane proteins such as occludins (82), claudins (81, 255), junctional adhesion molecules (25, 168), tricelluin (110), and intracellular scaffold proteins [such as zonula occludens (ZO) proteins ZO-1, -2, and -3] (98, 249) (Fig. 1). The role of specific TJ proteins on epithelial permeability has been shown in several knock-down and expression studies in polarized epithelial cell lines [reviewed in (81, 148, 252, 257)]. In the intestine, claudins -1, -3, -4, -5, and -8 decrease paracellular permeability, whereas claudin-2 forms cation-selective channels that decrease transepithelial permeability and reduce paracellular NaCl and water reabsorption (10). The ZO proteins, ZO-1, ZO-2, and ZO-3, all contain PDZ domains, which interact with other proteins including actin in the cytoskeleton (Fig. 1). ZO-1-deficient cells are still able to form TJs and display normal permeability, possibly due to the functional redundancy by ZO-2, but they have altered kinetics of TJ assembly (258).

Hyperpermeability of the intestinal barrier is believed to contribute to the pathogenesis of several gastrointestinal disorders including IBD, celiac disease, IBS, and food allergy (27). Inflammation associated with these diseases and disorders is likely to be one of the major inducers of TJ dysfunction and increased permeability. Several inflammatory cytokines including interferon-γ (IFN-γ) (2), TNF-α (164), IL-1β (9), and IL-17 (138) have been shown to cause increases in intestinal permeability through altered expression of TJ proteins, or increased expression of myosin light chain kinase (MLCK), which can alter TJ structure and paracellular permeability by phosphorylation of myosin II regulatory light chain (MLC) (250). In contrast, the anti-inflammatory cytokines such as IL-10 and transforming growth factor-β (TGF-β) enhance epithelial permeability and block the negative effects of infection with pathogenic *Escherichia coli* on epithelial permeability (105).

Epidermal growth factor has been shown to protect against the increased permeability caused by noxious stimuli including oxidative stress, ethanol, and acetaldehyde via MAPK activation and TJ modulation (23). Glutamine, an essential amino acid in pigs, was reported to enhance barrier function in vivo (277), and its absence in tissue cultures of Caco-2 cell monolayers, decreases expression of claudin-1 and increases transepithelial permeability (58, 157, 158).

TJ complexes and epithelial permeability are known to be affected by epithelial interaction with microbes and their metabolites. Studies in vitro have shown that stimulation of the Toll-like receptor 2 (TLR2) signaling pathway activates protein kinase C (PKC)-α and PKCδ, which in turn, lead to an increase in transepithelial resistance and a redistribution of ZO-1. Recently, administration of *Lactobacillus plantarum* to humans was shown to increase staining for ZO-1 and occludin in the vicinity of TJ structures in biopsy tissue (128). In vitro*, L. plantarum* also conferred protection against chemically induced disruption of the epithelial barrier in Caco-2 monolayers (128). TLR2 is expressed by epithelial cells (79) in vivo and recognizes diacylated or triacylated lipopeptides of bacteria and thus represents a plausible mechanism for the reported effects of probiotics on small intestinal barrier function.

As discussed below, the intestinal microbiota produce short-chain fatty acids (SCFAs), including butyrate, propionate, and acetate, which reach concentrations up to 100 mM in the colon due to the fermentation of complex carbohydrates. In vitro, low concentrations (2 mM) of butyrate were shown to increase transepithelial resistance and decrease inulin permeability in Caco-2 cell monolayers, whereas higher concentrations (8 mM) had an opposite effect, even inducing apoptosis in a concentration-dependent manner (197). In contrast, a recent study reported that 10 mM butyrate was shown to reduce the flux of 3-kDa FITC-dextran through Caco-2 monolayers compared with control cells, suggesting that it enhances intestinal permeability (133). Using a calcium switch assay to induce TJ formation, butyrate was shown to enhance TJ assembly, involving the AMP-activated protein kinase (197, 198).

## Mucus Glycoproteins

**Fig. 2.**
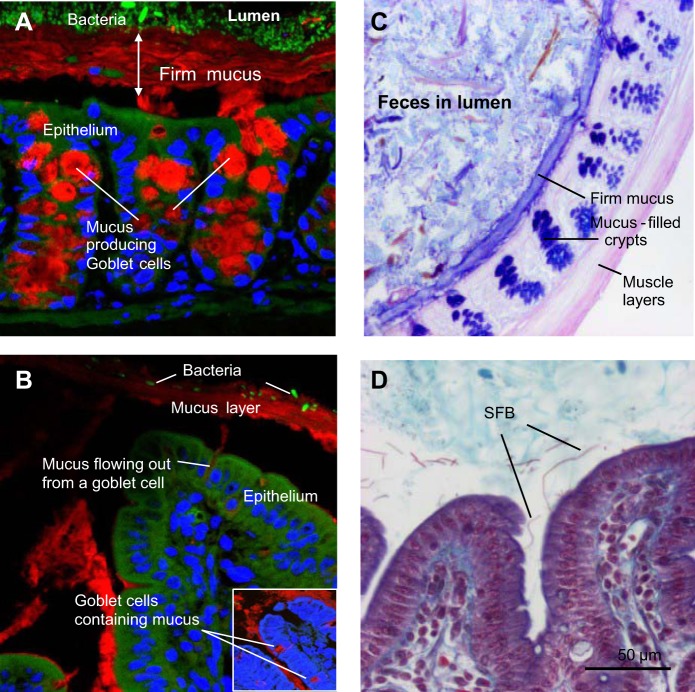
Fluorescence microscopy of mucus and microbiota in Carnoy-fixed sections of colon (*A*) and ileum (*B*) from mice. Mucin 2 (Muc2) was detected by immunofluorescence using anti-Muc2 and goat-anti-rabbit Alexa Cy3 antibodies (red). Nuclei were visualized using DRAQ5 (blue). Bacteria were identified using fluorescence in situ hybridization and the universal Euprobe 388 (green). *C*: Alcian blue/periodic acid Schiff-stained colonic tissue (frozen section) from a mouse showing a dark blue firm mucus layer, dark blue-stained goblet cells, and fecal material in the lumen. *D*: section of ileum (formalin fixed) from a conventional mouse stained with the Crossmon procedure. Arrows indicate segmented filamentous bacteria (SFB), which in contrast to other commensals, are typically found in contact with the epithelium.

Mucins, secreted by goblet cells in the epithelium, are the determining constituents of the mucus layer, which form a considerable physical barrier to enteric commensals and pathogens. The importance of the mucus glycoproteins for host protection is highlighted by the fact that absence of the main intestinal secreted mucin (MUC2) leads to spontaneous and lethal colitis (246, 265). The secreted mucins are glycoproteins, containing up to 80% carbohydrates in the form of a dense array of *O*-linked oligosaccharides, which are linked into a large macromolecular complex via cysteine-rich domains at both the amino- and carboxy-termini (57, 174). It is this extensive network structure that gives secreted mucus its viscous rheological properties. In humans there are five oligomerizing secreted mucins (MUC2, MUC5AC, MUC5B, MUC6, and MUC19), of which the first four are produced in different regions of the gastrointestinal tract (57). In the stomach the secreted mucus consists of MUC5AC and MUC6, which are produced by separate gastric mucous cells. Mucus in the small intestine is mainly composed of MUC2 and is produced by goblet cells (Fig. 2). In the small and large intestine the secreted mucus is predominantly composed of MUC2, although in the large intestine, MUC5AC and MUC6 may be produced in small quantities under some conditions (174).

In humans, the goblet cell-to-enterocyte ratio increases from the proximal to distal intestine, with an estimated 4, 6, 12, and 16% of goblet cells in the epithelium of the duodenum, jejunum, ileum, and distal colon, respectively (137). Mucus thickness varies at different intestinal locations and can be studied by applying charcoal particles to mounted tissue explants and microscopically measuring the transparent gap between the particles and the surface of the epithelium (17). In rats the mucus layer was thickest in the colon (~830 μm) and thinnest in the jejunum (~123 μm). Aspiration of the loose mucus from the apical surface leaves a “firm” mucus layer adhered to the epithelium (17). In the rat colon the firm mucus layer was ~116 μm thick but only ~20 μm or absent in the small intestine. In the ileum, mucus with a long, sticky, rope-like structure flows above the villi spatially compartmentalizing the bacteria to the lumen (Fig. 2*B*). The exception is segmented filamentous bacteria that lie beneath the mucus strands in the small intestine and attach themselves to epithelial cell (55). This overall structure of the intestinal mucus layer in small and large intestines is conserved in rats, mice, and humans.

Carnoy fixation and paraffin embedding technique prevents complete shrinkage of the mucus and has also been used to assess mucus thickness (174) (Fig. 2). Immunofluorescent visualization of mucus and microbiota in Carnoy-fixed tissue showed that the firm mucus layer in the colon is largely devoid of intact bacteria (120) (Fig. 2*A*). The secreted mucus contains several secreted host factors including trefoil peptides, and antimicrobial factors such as regenerating islet-derived protein 3 (Reg3) proteins and secretory IgA, which play an important role in the immune exclusion of microorganisms and other antigens to the mucosal surface (see below). A recent study has revealed that the composition of the gut microbiota can influence the penetrability of the firm mucus with genetically identical animals housed in the same facility differing with respect to the penetrability of the colonic mucus (116). The colony of mice with a mucus layer that was penetrable to bacteria had relatively higher levels of Proteobacteria and TM7 bacteria in the distal colon mucus than the colony of mice with an impermeable mucus barrier. Gnotobiotic mouse models have also been used to study the influence of two major commensal bacteria, *Bacteroides thetaiotaomicron* and *Faecalibacterium prausnitzii*, on the intestinal mucus layer. *B. thetaiotaomicron* increased goblet cell differentiation and mucin synthesis, but when associated with *F. prausnitzii* these effects were diminished (293). Recently, it was shown that colonic mucus remains permeable to bacteria-sized beads for 6 wk following colonization of germ-free mice with conventional mouse microbiota (116). These changes in mucus properties correlated with changes in the development of microbiota ecosystem, suggesting that similar changes might be observed after weaning.

Although secreted mucin is expressed constitutively by goblet cells, its production is upregulated by TLR signaling to replenish that degraded by commensals or removed by peristalsis (109). Additionally, IL-22, a cytokine produced by type 3 innate lymphoid cells and Th17 cells, stimulates MUC1 production and the enhancement of epithelial regeneration with goblet cell restitution (245) [reviewed in (181)]. A broad range of cytokines, including some produced by epithelial cells, can also influence mucin production [reviewed in (174)]. Recently, butyrate was shown to stabilize hypoxia-inducible factor in vivo (133), a transcription factor that regulates metabolism and other aspects of intestinal barrier function, including mucin production (161).

Intestinal epithelial cells produce transmembrane mucins, which are crucial components of the glycocalyx on the apical surface of mucosal epithelium. The cell-surface mucins produced in the human gastrointestinal tract include MUC1, MUC3A, MUC3B, MUC4, MUC12, MUC13, MUC15, MUC16, and MUC17 (99), and their expression varies at different locations along the gastrointestinal tract. The cell-surface mucins are considered to be cleaved during biosynthesis resulting in a smaller membrane-attached part that is joined to the larger secreted component via a conserved sea-urchin sperm domain (36, 99). Like the secreted mucins, the mucins forming the glycocalyx are extensively *O*-glycosylated on the extracellular domains. Apart from steric hindrance of bacterial binding the cell–surface, MUC1 can modulate nuclear factor-κB signaling through its cytoplasmic domain (6). Pathogen binding also enhances shedding of cell-surface mucins as a mechanism to release pathogens from the surface. In this context it is important to note that the oligosaccharides found on cell-surface mucin may mimic ligands for microbial adhesins (101).

## Secretory IgA

Humans secrete an estimated 3 g of secretory immunoglobulin A (sIgA) into the intestinal lumen every day, reflecting its important role in protecting the mucosal surface. Intestinal sIgA levels in germ-free mice increase soon after colonization with bacteria, as does the number of sIgA-secreting plasma cells in the LP. Around 25 to 75% of sIgA is reported to bind to the commensal microbiota, suggesting that it also shapes the composition of the microbial community (123). The important contribution of sIgA to barrier function is evident from studies in B cell-deficient mice and in mice lacking the polymeric immunoglobulin receptor required for sIgA transport to the lumen. Both of these knockout mice have enhanced stimulation of innate responses in gut epithelial cells in the small and large intestines (240). Further evidence for the role of adaptive immune responses in controlling mucosal inflammatory responses to commensal bacteria comes from studies in mice that lack a functional adaptive immune system. In these immune-deficient mice, bacterial colonization results in a stronger intestinal innate response than their wild-type counterparts (40, 132), demonstrating that the adaptive immune response contributes to minimizing activation of the innate immune system by the gut microbiota. Secretory IgA levels are normal in mice lacking CD40, a receptor that on B cells mediates “T cell help” and also in humans lacking germinal centers, suggesting the existence of T cell-independent pathways of IgA induction. Both T cell-dependent and -independent mechanisms of IgA induction and their contribution to the IgA pool and its specificity have been recently reviewed (193) and are not discussed here in detail.

**Fig. 3.**
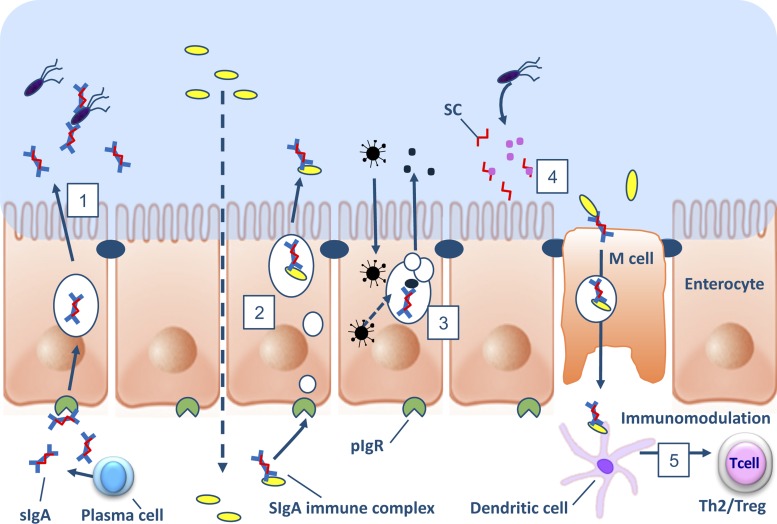
Schematic representation of the protective mechanism of IgA, secretory IgA (sIgA), or secretory component (SC) in the intestinal mucosa. *1*: plasma cells in the lamina propria (LP) produce polymeric IgA, which is transported across epithelial cells (a process known as transcytosis) to the lumen by the polymeric Ig receptor (pIgR), where it may interact with antigens of bacteria, viruses, toxins, etc. to exclude them from contact with the epithelium. *2*: in the LP, polymeric IgA (pIgA) can bind to immune complexes, including those comprising infectious agents, leading to their removal by removed by transcytosis. *3*: pIgR-mediated trafficking of pIgA through epithelial cells can interfere with intracellular viral assembly in the Golgi apparatus. *4*: free SC in the lumen has been shown to neutralize pathogen-derived toxins and adehsins. *5*: sIgA facilitates uptake of pathogens into IgA-inducing Peyer’s patches and isolated lymphoid compartments and presentation to dendritic cells the subepithelial dome region. Recognition of sIgA by dendritic cells is reported to inhibit IL-12 cytokine secretion, leading to induction of helper T cell 2 (Th2) or regulatory T cell (Treg) responses.

A role for epithelial TLR4 signaling in B cell recruitment and IgA class switching was demonstrated in transgenic mice expressing a constitutively active form of TLR4 in intestinal epithelial cells (237). These mice had substantial increases in B cell recruitment to the mucosa and IgA production that was linked to increased intestinal epithelial expression of the chemokines CCL20, CCL28, and cytokine APRIL, a potent B cell activator. In the large intestine, pattern recognition receptor signaling also appears to induce IgA class switching to IgA2, which is more resistant to proteolysis through the increased expression of APRIL and BAFF in intestinal epithelial cells (100) (Fig. 3). Interestingly, TLR signaling has additionally been reported to enhance uptake of particulate antigens in Peyer’s patches (PP), suggesting that induction of sIgA antibody responses might be directly modulated by the extent of microbial signaling in the follicular epithelium (41). Recently, the microbiota of low-IgA mice were shown to vertically transmit an IgA-low dominant phenotype to genetically identical mice in the same facility (183). These findings were shown to be a result of the degradation of the secretory component of sIgA as well as IgA itself by bacteria from low-IgA mice. Moreover, these results highlight the fact that when comparing wild-type and mutant mice from different facilities and breeders, microbiota exposure should be equivalent to minimize nonchromosomal phenotypic variation (183).

Secretory IgA produced by plasma cells in the mucosal LP is recognized by the polymeric Ig receptor (pIgR) expressed on the basal membrane of enterocytes. Binding of sIgA to pIgR through the secretory component results in transport through vesicles and release into the lumen, a process known as transcytosis (30) (Fig. 3). The secretory component (SC) in sIgA confers hydrophilic properties to the Fc fragment of the IgA antibody, which is considered important for interaction with mucus, and therefore proper anchoring in the secreted mucus layers. The primary mechanism of sIgA-mediated protection is immune exclusion, referring to the antibody binding to microorganisms and toxins, thereby preventing colonization or toxicity and damage to epithelial cells (177) (Fig. 3). Compelling evidence for this mechanism of protection comes from several animal and in vitro models (11, 28, 108, 201, 213, 241, 287), including studies using a hybridoma implanted in the back of mice that secretes antigen-specific IgA (33, 292). Additionally, sIgA can bind to intracellular pathogens in endosomes during transcytosis to the lumen (Fig. 3). This mechanism was shown to inhibit key steps in the assembly of influenza, Sendai virus, and rotavirus, and contribute to immunity capable of infecting mucosal epithelial cells (48, 78, 171, 221). Furthermore, sIgA may also bind to antigen complexes formed in the LP before pIgR-mediated transport through epithelial cells, thereby reducing the likelihood of inflammatory reactions and systemic responses (124, 215) (Fig. 3). The free SC, a polypeptide comprising the extracellular portion of the pIgR that remains attached to dimeric IgA after transcytosis, is also reported to have protective functions at the epithelial surface. The free SC released in secretions has been demonstrated to neutralize *Clostridium difficile* toxin A and enteropathogenic *E. coli* intimin via interaction with sialic and galactose residues present on the SC polypeptide (200) (Fig. 3).

Apart from its direct effects on immune exclusion of pathogens and pathogenicity factors, sIgA is reported to contribute to homeostasis by promoting anti-inflammatory responses at mucosal surfaces. In the small intestine sIgA facilitates uptake of pathogens into IgA-inducing Peyer’s patches and isolated lymphoid compartments (122), and recognition of sIgA by dendritic cells is reported to inhibit IL-12 cytokine secretion, leading to induction of helper T cell 2 (Th2) or regulatory T cell (Treg) responses (24, 150) (Fig. 3). These functions of sIgA collectively reinforce the integrity of the intestinal barrier, dampen proinflammatory immune responses, and thereby contribute to intestinal homeostasis.

Despite the recognized importance of sIgA in gut barrier protection, it should also be mentioned that polymeric IgM actively transported across epithelia by pIgR as well as IgG from local secretions, can also contribute to protection of mucosal epithelium (39, 70, 89, 185). This is partly the explanation for why deficiencies in IgA in both humans and mice do not result in chronic inflammation (97), but also because defects in the production of IgA can be largely compensated by other gut barrier functions, including the range of antimicrobial products produced by epithelial cells. Many individuals affected with IgA deficiency have no apparent symptoms, whereas others suffer from recurrent mucosal infections, allergies, and autoimmune disease (4).

## Antimicrobial Peptides and Proteins

The intestinal epithelium produces and secretes a vast array of antimicrobial peptides and proteins (AMPs) into the lumen that contribute to the multilayered defense against luminal microorganisms (Table 1). Growing preclinical and clinical evidence supports an essential role for these molecules in gut protection against infection and inflammatory disease. Besides their direct bactericidal activity, AMPs represent a link between innate and adaptive immunity. The aim of this section is to summarize the available evidence about the role of AMPs in host defense, and the mechanisms by which the intestinal antimicrobial response is regulated. The structure-function relationship of mammalian antimicrobial peptides has been reviewed recently (54, 84, 115, 184, 283) and is not discussed in detail here.

**Table 1.** Human and mouse antimicrobial peptides and proteins produced in intestinal epithelium

Human	Mouse	Epithelial-Producing Cells	Expression Regulation	Biological Activities	References
hBD1	mBD1	Enterocytes in SI and colon	Constitutive	Antimicrobial (gram-positive bacteria, fungi), chemotactic	189
hBD2, 3, 4, 5, 6	mBD2, 3, 4, 5	Enterocytes in SI	Upregulated by infection and inflammation	Antimicrobial (gram-positive and negative bacteria, fungi), chemotactic	283
HD5, HD6	Cryptidins	Paneth cells in SI	Constitutive	Antimicrobial (HBD5: gram-positive and negative bacteria, fungi), entrapment (HBD6)	283
Cathelicidin (LL37)	CRAMP	Enterocytes in colon	Upregulated by butyrate	Antimicrobial (gram-positive and negative bacteria, fungi), chemotactic	54, 283
Lysozyme C	Lysozyme C	Paneth cells and enterocytes in SI	Constitutive	Antimicrobial (gram-positive bacteria)	126, 184, 206
BPI	BPI	Enterocytes	Constitutive, upregulated by anti-inflammatory eicosanoids	Antimicrobial (gram-negative bacteria), LPS binding	35, 37, 38, 54
sPLA2		Paneth cells	Constitutive	Antimicrobial (gram-positive bacteria), eicosanoide metabolism	54, 184
HIP/PAP (Reg3α)	Reg3β	Paneth cells and enterocytes mainly in SI	Upregulated by infection and inflammation	Antimicrobial (gram-negative bacteria), bacterial entrapment	179, 262
	Reg3γ	Paneth cells and enterocytes mainly in SI	Upregulated by infection and inflammation	Antimicrobial (gram-positive bacteria), bacterial entrapment	40, 160
	ANG4	Paneth cells	Upregulated by commensals and pathogens	Antimicrobial (gram-positive and negative bacteria, nematodes), angiogenesis	104
Elafin	Elafin	γδT cells, goblet cells	Upregulated by LPS, inflammation, and defensins	Anti-proteases, antimicrobial (Gram positive and negative bacteria, protozoa, viruses)	73, 223

ANG4, angiogenin 4; BPI, bacterial permeability increasing protein; hBD, human β-defensin; HD, human α-defensin; HIP/PAP, hepatocarcinoma-intestine-pancreas/pancreatic associated protein; LPS, lipopolysaccharide; Reg3, regenerating gene family protein 3 (islet-derived); SI, small intestine; sPLA2, secretory group IIA phospholipase.

Virtually all the epithelial cell types in the intestine can produce AMPs. However, the largest amounts of AMPs are produced by enterocytes lining the gastrointestinal tract and by Paneth cells in the small intestine. Expression of some Paneth cell antimicrobials varies along the small intestine, with the highest amounts produced in the ileum (129, 279). Paneth cells are absent in the large intestine, which leads to a different AMP expression profile in the small and large intestines, although intact Paneth cell products have been detected in the colonic lumen (169) and they possibly contribute to the colonic antimicrobial background. Therefore, the antimicrobial pressure is likely to change along the gut and this may play a role in shaping the distinct microbiota profile observed in the different segments of the intestine. AMPs appear to be concentrated close to the epithelium and within the firm layers of mucus, which may account for the relatively high numbers of bacteria found in the lumen (178).

Expression of different groups of AMPs appears to be regulated by diverse mechanisms. Some groups, including most alpha-defensins, are constitutively expressed and do not require microbiota signaling, as shown by comparing their expression levels in germ-free and conventional animals (207, 283). In contrast, innate recognition of microbes via TLRs and nucleotide oligomerization domain (NOD)-like receptors can upregulate expression of other AMP groups, including the human β-defensins hBD2 and hBD3 (189, 280), ANG4 (104), and the C-type lectins Reg3β (210) and Reg3γ (40) or resistin-like molecule beta (RELM-β) (12, 210). Accordingly, Nod2^−/−^ (141), MyD88^−/−^ (a TLR signaling adaptor) (260), and TLR2^−/−^ mice (176) display lower amounts of different Paneth cell antimicrobials than wild-type mice. Apart from regulation of AMP expression, signaling through innate receptors also controls secretion by Paneth cells. This was first demonstrated by Ayabe and colleagues (18) by triggering isolated small intestinal crypts with different groups of microbes and microbial molecules. Exposure to intact bacteria and bacterial products induced a fast Paneth cell degranulation and antimicrobial activity release. By contrast, fungi and protozoa do not stimulate antimicrobial secretion, suggesting that Paneth cells are primarily involved in intestinal protection against bacteria.

Recent studies in animal models indicate a key role for AMPs in limiting the access of luminal bacteria to the mucosal surface. Meyer-Hoffert et al. (178) showed that AMPs secreted in the small intestine are essentially retained by the mucus layer covering the epithelium, and only minor levels of these molecules are found in the lumen. These data suggest that high AMP concentration in the mucus coat can contribute to limit the bacterial load associated with the epithelium without exerting excessive pressure on the luminal microbiota. Accordingly, research directed by Hooper (260) showed that the presence of functional Paneth cells was essential to control levels of bacteria associated with the epithelium and to limit the translocation of both pathogenic and commensal bacteria through the intestinal barrier. In contrast, no changes in total numbers of luminal bacteria were observed in animals with defective Paneth cell function (260). Recently, both MyD88 and Reg3γ were shown to play a role in spatially separating the microbiota from the epithelium without any detected effect on the number and species profile of luminal communities (160, 261). Interestingly, in agreement with the reported antimicrobial specificity of this lectin, only the numbers of gram-positive, but not gram-negative, mucosa-colonizing bacteria were increased in Reg3β-deficient mice (261). Further evidence for the role of Reg3 polypeptides in intestinal defense against pathogens comes from studies in Reg3β-deficient mice, which were shown to be more susceptible to infection than wild-type mice. Enteric challenge of Reg3β-deficient mice with *Yersinina pseudotuberculosis* (60) or *Salmonella enteritidis* (262), leads to increased mucosal colonization and translocation by these pathogens, without affecting their survival in the lumen. The effect of Reg3β seemed to be specific to gram-negative bacteria because there was no effect of the knockout on mucosal colonization and translocation of the gram-positive pathogen *Listeria monocytogenes* (262). Reg3γ was recently shown to have a protective role against mucosal infection with pathogenic *Listeria* and *Salmonella* in vivo (160). These results suggest that a complementary activity of these two closely related mouse Reg3 proteins exists in vivo. Additional evidence for the importance of Paneth cell AMPs in regulating microbiota was demonstrated in mice that overexpress human alpha-defensin 5 (HD5) or that lack matrix metalloproteinase 7 (MMP7), which is required for the proteolytic activation of alpha defensins (225). HD5 overexpression resulted in eradication of segmented filamentous bacteria (225), a commensal tightly associated to the ileal epithelium and known to play an important role in the development and regulation of mucosal immunity (114). Mmp7^−/−^ mice had an altered microbiota profile compared with wild-type mice, but the total numbers of luminal microbiota were not affected as was described for Reg3γ^−/−^ mice.

By limiting the interaction and penetration of bacteria through the intestinal mucosa, AMPs might be expected to play an important role in the pathogenesis of inflammatory and infectious diseases. Evidence to support this notion is provided by a series of studies involving transgenic animals and human individuals with defective AMP levels or Paneth cell function, or both. For example, an in vivo antiparasitic function of RELM-β has been shown in mice genetically deprived of this goblet cell peptide (102). Also, cathelicidin knockout mice had increased susceptibility to *E. coli* O157:H7 infection (45), whereas Mmp7^−/−^ mice were more sensitive to chemically induced colitis (238) and to pathogen infection (291) than wild-type animals. Conversely, mice overexpressing the human alpha-defensin HD5 (224) displayed increased resistance to infection by *Salmonella typhimurium*. In line with these findings, a decreased expression of HD5 in jejunal mucosa was associated with increased susceptibility to infectious diarrhea in a human cohort (134), and reduced expression of LL-37 and of hBD-1 was reported in adults and children during the early phases of *Shigella* infection (111). A number of clinical reports also support a role for AMPs in preventing chronic intestinal inflammation. For instance, single nucleotide polymorphisms of the *hbd-1* gene are strongly associated with colonic Crohn’s disease (142), whereas defective expression of alpha-defensins (281) and of hBD-1 (280) has been observed in this condition. Interestingly, low copy numbers of the gene encoding for hBD-2 and reduced mucosal expression of hBD-2 in healthy individuals have been identified as a risk factor for Crohn’s disease (69). Downregulation of *hbd-1* and *hbd-4*, has also been reported in duodenal biopsies of celiac disease in pediatric patients (272). Furthermore, mutations in a series of genes involved in Paneth cell differentiation and function have been identified as risk factors of ileal Crohn’s disease. Some examples include TCF-4 (143), a transcription factor involved in Paneth cell differentiation (204); NOD2 (107), a cellular receptor for bacterial motifs mediating the Paneth cell response to luminal bacteria (141, 202); and ATG16L1 (222), which is involved in Paneth cell granule exocytosis and phagosomal killing of invading bacteria (34, 222).

## Gut Microbiota and Barrier Function

The intestinal tract harbors one of the densest and most complex microbial ecosystems associated with mammals and humans. In the small intestine, the number of microorganisms is relatively low compared with that of the colon, and in the ileum they reach densities of 10^7^ to 10^8^ cells per milliliter of contents (95, 236). The human large intestine is larger in diameter than the small intestine; does not contain villi; and in humans includes the cecum and the ascending, transverse, and descending colon. Here most of the microbes are found with densities of 10^10^ to 10^11^ cells per milliliter of contents (155, 236). A detailed characterization of the microbiota along the gastrointestinal tract and its variation over time has been recently described (52, 208, 236, 248, 297). Two major phyla, Bacteroidetes and Firmicutes, dominate the microbiota of humans, and although their abundance in fecal samples remains relatively constant in healthy subjects, many studies have shown the considerable inter- and intrapersonal variability at the genus level and above (63, 155, 156, 208). Previously, the gut microbiota was estimated to consist of 500–1,000 species of microbes (220), but a recent large-scale study has estimated that the collective human gut microflora is composed of more than 35,000 bacterial species (77). The MetaHIT consortium (13) proposed the concept of intestinal enterotypes in humans reflecting three different host-microbial symbiotic states that are defined by the dominance of Bacteroides, Prevotella, or Ruminococcus. Alternative interpretations of the enterotype concept have also been proposed suggesting that that continuous variation of the human microbiota diversity is a better-supported conclusion (140). Gut microbial community composition varies less within an individual than among different individuals, suggesting a strong environmental component (52, 66, 234). Examples of environmental factors influencing microbiota composition include age, geographic location, dietary habits, and antibiotic use [see (167, 190, 259)].

Symbioses with intestinal microorganisms is known to have a profound effect on the mammalian physiology by, for example, influencing tissue and immune development (127, 144, 243), providing metabolic functions (19, 247), and providing colonization resistance against pathogens [reviewed in (32)]. However, the beneficial effects of the gut microbiota are highly dependent on its composition, which has been shown to change dramatically in several human disorders and diseases (77, 130, 231) [also see recent review (236)]. For some diseases the altered composition or emergence of pathobionts may contribute to the pathophysiology of a disease, as shown in Crohn’s disease (56, 166, 232), metabolic diseases such as type II diabetes (146), and obesity (42, 226).

Much of the knowledge about microbiota composition mentioned above has been generated from fecal material, particularly studies of human microbiota. However, some studies have shown distinct mucosal populations within the mucosal and luminal niches within healthy individuals (214). For example, segmented filamentous bacteria colonizes the epithelium in the ileum and is found beneath the detached mucus layer (55). A few studies have shown that the mucosa-associated microbiota differs substantially from the luminal microbiota (63, 274, 290). Most bacteria are restricted to the lumen, but some, such as *Bacteroides fragilis*, was shown to colonize both the lumen and crypts of the colon (152). The mucosa-associated bacteria, including mucin-degrading specialists, are scattered among the gut microbiota-associated phyla and include species that can degrade mucins such as *Akkermansia muciniphila*, *Bacteroides thetaiotaomicron*, *Bifidobacterium bifidum*, *B. fragilis*, *Ruminococcus gnavus*, and *Ruminococcus torques* (205, 214). It is likely that the community of mucosal-associated bacteria are those that promote mucus secretion and increase mucus thickness through release of microbe-associated molecular patterns (MAMPs) and the production of SCFAs (21, 242, 289). Adherence to the mucus can allow these species to outcompete others depending on the rates of production and release of the mucus (235).

As mentioned under *Antimicrobial Peptides and Proteins*, microbiota play a key role in influencing epithelial barrier functions through their interactions with innate pattern recognition receptors, particularly the TLRs and NOD-like receptors (NLRs) (1, 285, 286). These innate receptors recognize common MAMPs such as lipopolysaccharide (LPS), which binds to TLR4. It may not be necessary for microbes to colonize the epithelial cell surface to trigger TLR signaling because MAMPs are also released from both live and dead microbes in the intestine. Bacterial MAMPs may differ in their capacity to trigger TLR signaling depending on the species. Of particular importance is the recent finding that that LPS from *Bacteroides dorei* harbored tetra- and penta-acylated lipid A structures, as opposed to the hexa-acylated lipid A observed in *E. coli*. Moreover, the presence of *Bacteroides* species in the microbiota of children in countries with high susceptibility to autoimmunity produce a type of LPS with inhibited immune stimulation and inflammatory signaling (270), which was associated with increased incidence of type 1 diabetes. It was hypothesized that the immune inhibitory properties of *Bacteroides* LPS may prevent early education of the mucosal immune system and contribute to the development of type 1 diabetes.

Accumulating evidence shows the importance of TLR and NLR signaling to homeostasis in the intestine (186, 209). Intestinal epithelial cell-specific deletion of TLR4, the TLR signaling adaptor protein MyD88, and NOD1 in mice, leads to impaired immunity to bacterial infections (149, 233). TLR5 knockout mice have a tendency to develop spontaneous colitis due to a failure to control translocation of the microbiota (147). NOD2 polymorphisms in patients with Crohn’s disease are associated with decreased intestinal defenses via reduced secretion of antimicrobial proteins and intracellular killing of microbes (107, 165). Additionally, patients with Crohn’s disease with NOD2 polymorphisms have reduced numbers of intestinal LP Tregs due to the role of NOD2 signaling in promoting survival of human regulatory T cells.

As mentioned above, TLR signaling is involved in IgA production, maintenance of TJs, and expression of antimicrobial peptides, all functions that are crucial to maintaining an intestinal barrier (4, 85, 96, 159, 176, 118a, 260, 271). Despite these clear beneficial roles, chronic proinflammatory responses involving immune cells in the LP need to be avoided to prevent barrier destruction and pathology. Thus, several host mechanisms exist to tightly regulate inflammatory signaling in response to the microbial threat (1, 79, 151, 191, 216, 218, 239). Additionally, the microbiota considered to contribute to the maintenance of homeostasis, a prominent example being *F. prausnitzii*, which has anti-inflammatory activities that attenuate colitis development in mouse models (217, 219, 244). Many studies have shown that the relative abundance of this normally abundant colonic species is reduced in patients with active IBD and thus may contribute the loss of homeostasis and inflammatory pathology (180).

Segmented filamentous bacteria are found in the gut of many vertebrate species, including mice and possibly humans (139), and have gained much attention due to their firm attachment of the growing filaments to epithelial cells and their capacity to stimulate innate immunity, IgA responses, and striking increases in small intestinal Th17 cells (83, 112). Moreover, mice that acquire segmented filamentous bacteria have enhanced small intestinal and pulmonary type 17 immunity and enhanced resistance to *Staphylococcus aureus* pneumonia, and the intestinal pathogen *Citrobacter rodentium* (87, 112). Thus, manipulation of this commensal-regulated pathway may provide new opportunities for enhancing mucosal immunity. More recently, a strong correlation was observed between adhesion and Th17 cell induction via the induction of a Th17-inducing program in the epithelium (14).

The effects of some bacterial species on the epithelial barrier and immune response have been characterized in germ-free mice or in vitro studies. These include (model) commensals such as *Bacteroides* spp., *F. prausnitzii* (244), *Akkermansia muciniphila*, *Roseburia* spp.; probiotic bacteria including *Bifidobacterium* and *Lactobacillus* spp.; or specific pathobionts such as *Helicobacter*; or bacteria belonging to Enterobacteriaceae (e.g., *E. coli*, *Citrobacter)*. The emerging picture is that the responses are specific for each microbe studied, but further comparative work is needed to substantiate this and to discover general patterns. Moreover, the responses relate to animal and in vitro models and must be translated to the human situation. Pioneering studies using human volunteers and probiotic candidates has opened up the possibility to investigate mucosal transcriptional responses to specific bacteria (263, 264) and modification of small intestinal TJs (128).

Many of the physiological effects of the microbiota can be attributed to end products of fermentation, SCFAs (primarily acetate, propionate, and butyrate), branched-chain fatty acids (isovalerate, isobutyrate, and caproate), H_2_, CO_2_, and CH_4_. Acetate is the most abundantly produced SCFA in the colon and its production is a common feature of most gut microbiota members. Propionate is more restricted to Bacteroidetes, *Clostridium* cluster IX (*Veillonella*), *Clostridium* cluster XI (*Megasphaera*), and Actinobacteria (*Propionibacterium*). Butyrate production is generally restricted to some *Clostridium* clusters (IV and XIVa) from the Firmicutes phylum. Besides acetate, lactate can also be a precursor for butyrate production underpinning the notion of metabolic cross-feeding between gut bacteria. Loss of some bacteria members will disturb this interaction and will undeniably alter the abundance or ratio of SCFAs, and subsequently, interaction with the host. Butyrate has been the most studied SCFA for its pleiotropic effects on metabolism, immune function, and epithelial barrier. Butyrate exerts various beneficial effects in the host such as enhancement of intestinal barrier function in vitro using cell lines (198), reduction of translocation of *E. coli*, and attenuation of visceral pain [for a review see (93)]. Recently, administration of butyrate-producing *Clostridium tyrobutyricum* was shown to prevent acute dextran sodium sulfate-induced colitis in mice (106). Administration of the spore-forming component of indigenous intestinal microbiota, particularly clusters IV and XIVa of the genus *Clostridium*, was shown to promote Treg accumulation in the LP of the colon (16). Mucosal Tregs play a key role in maintaining an anti-inflammatory tone in the gut and thus in the preservation of an intact barrier. A follow-up study showed this could also be achieved with a more restricted population of *Clostridium* and oral inoculation during the early life of conventionally reared mice resulted in resistance to colitis and systemic IgE responses in adult mice (15). Shortly after that study, butyrate was shown to induce the differentiation of colonic Treg cells in mice via increased epithelial expression of TGFβ (80).

Although the intestinal microbiota is remarkably stable over time, its equilibrium and symbiotic homeostasis with the host can be disturbed (i.e., dysbiosis). Many human disorders have been linked to an altered microbiota composition with reduced diversity and lack of butyrate-producing bacteria in comparison to healthy individuals. Such perturbations are frequently associated with immune and metabolically related diseases. Whether this disturbance in the microbial community is the cause or effect of a loss in the homeostatic relation with the host remains to be determined. Nevertheless, there is good evidence that an altered microbiota can contribute to the pathophysiology of some diseases (44).

Another function of the commensal microbiota is the antagonism of pathogens through the production of bacteriocins or through competition for nutrients, commonly known as colonization resistance (32). The protective function of a healthy microbiota is clearly evident from antibiotic administration, which can sometimes result in intestinal problems such as antibiotic-associated diarrhea caused by enteropathogens and dysregulation of intestinal homeostasis (61, 173).

## Gut-Brain Axis and Immune System—An Interdisciplinary View of Gut Barrier Function

**Fig. 4.**
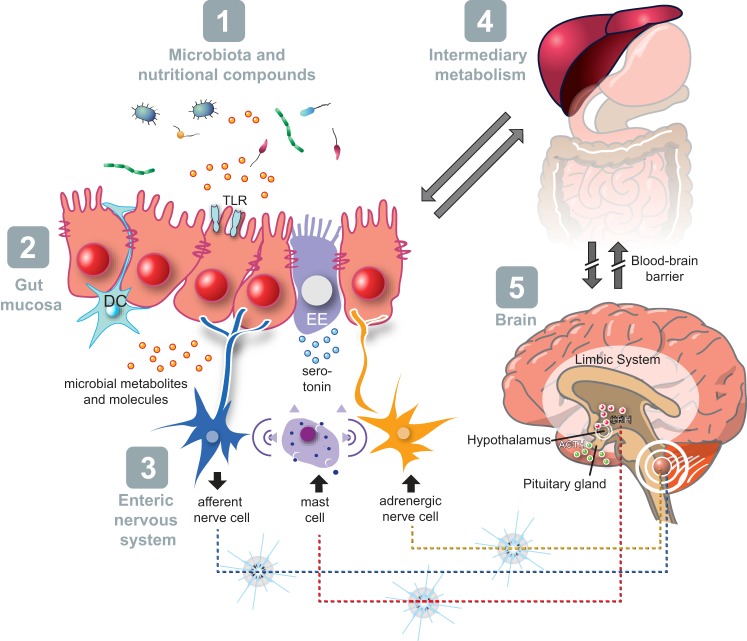
Schematic model of gut-brain signaling representing five components, with a central role of host-microbe interaction and intestinal barrier function (for detailed description, see text).

In this section we describe the functional evidence for bidirectional signaling between the central nervous system and enteric nervous system, linking neurological activity in the different parts of the brain with peripheral intestinal functions. The integrated model of bidirectional gut-brain signaling has five major components [i.e., intestinal microbiota, intestinal epithelium, enteric nervous system, intermediary metabolism, and the brain (Fig. 4)]. Important mediators of this bidirectional signaling include serotonin (5-HT), other monoaminergic, opioid, and endocannabinoid compounds, the autonomic nervous system, hypothalamus-pituitary-adrenal (HPA) axis, gut hormones, cytokines, and other gut-derived metabolic signaling molecules (e.g., growth factors). Serotonin, which is affected by intermediary metabolism, plays a key role in this gut-brain signaling (135, 136, 145). Functional evidence of efferent communication between the central nervous system and the gut mucosa has been widely reported. Extrinsic afferents include the vagal nerve and pelvic parasympathetic nerves, and postganglionic sympathetic neurons. These could also act via axon connections to other intrinsic enteric neurons. The stress associated with separation of neonatal mice from their mothers induces intestinal hyperpermeability due to increased secretion of corticotropin-releasing factor from the hypothalamus leading to release of the neurotransmitter acetylcholine by cholinergic neurons in the submucosa of the intestine (86). However, other animal studies report that vagus nerve activity can be protective in maintaining gut barrier function and TJ integrity under pathological conditions. In burn-induced intestinal injury, vagal nerve stimulation attenuated burn-induced intestinal hyperpermeability through activation of enteric glial cells (EGCs) (51). Emerging evidence underlines a major role of EGCs in the regulation of intestinal barrier function. EGCs inhibit intestinal epithelial cell proliferation and decrease intestinal paracellular permeability (267). The protective effect of EGCs on intestinal hyperpermeability involves EGC-derived S-nitrosoglutathione, as has been shown in a model of epithelial barrier defect induced by *Shigella flexneri* (74). Neuroimmune communications involving the vagus nerve have been shown to have a profound effect on intestinal barrier function. First, an anti-inflammatory effect of vagal afferences (90% of vagal fibers) has been shown (172). More recently it has been shown that vagal efferences (10% of vagal fibers) also play an anti-inflammatory role via acetylcholine, which is able to inhibit cytokine release directly via the α7 nicotinic acetylcholine receptor expressed on macrophages (276). The cholinergic anti-inflammatory pathway may also involve an indirect modulation of innate inflammatory processes via postganglionic modulation of immune cells in primary immune organs [i.e., spleen (254)].

Concordant to the concept of the microbe-gut-brain axis, not only is intestinal function affected by the brain, but also brain function may be influenced by intestinal factors including that of its microbial community (31, 72), possibly partly by the vagal route (266).

The intestinal barrier function plays a pivotal role in bidirectional gut-brain signaling and associated immune function. Deterioration of the intestinal barrier and deregulation of immune responses are associated processes and may provoke mucosal inflammation and increased afferent sensory signaling leading to abdominal complaints (7). Animal studies have elegantly provided evidence for the relationship between intestinal barrier impairment, mucosal immune activation, and visceral sensitivity. Accordingly, Su et al. (250) have developed transgenic mice that express constitutively active MLCK specifically within intestinal epithelia. Application of an acute stress in rats led to increased gut paracellular permeability and visceral sensitivity. Interestingly, prevention of stress-induced gut permeability using a TJ blocker abolished visceral hypersensitivity (7), suggesting that gut hyperpermeability is responsible for visceral hypersensitivity. A positive correlation between increased gut permeability and hypersensitivity to visceral nociceptive stimuli has also been demonstrated in patients with IBS (298). Besides the interplay with visceral sensory pathways, prevention of gut leakiness also leads to an attenuated HPA-axis response to stress. In a model of acute stress, blockade of epithelial cell cytoskeleton contraction by an inhibitor of MLCK activation or by a probiotic, *Lactobacillus farciminis*, was able to decrease MLC phosphorylation (8), resulting in suppressed stress-induced hyperpermeability, endotoxemia, central neuroinflamamtion, and attenuated HPA-axis response to stress (8). Attenuation of the HPA-axis response to stress has also been observed by use of antibiotic treatment to lower intestinal levels of LPS. These results taken together suggest that intestinal barrier impairment and subsequent decrease in LPS translocation are primary causes of the HPA-axis response to acute stress.

Brain function affects intestinal barrier function as clinically illustrated by increasing evidence that depression and (psychological) stress is associated with exacerbations of IBD and with the pathogenesis of IBS (88, 153, 170). This may be explained by serotonergic deregulation and the fact that psychological and physical stress are associated with deterioration of intestinal barrier function via mast cell-dependent (275) and mast cell-independent mechanisms (59). Recently, the role of microbial metabolites such as butyrate has gained interest in relation to improving human colonic function under stressed conditions (92), and butyrate may ameliorate bacterial translocation through stressed epithelium (154).

## Biomarkers of Intestinal Epithelial Permeability and Integrity in Blood, Feces, or Urine

Measurements of intestinal permeability are often used synonymously with the term “gut barrier function,” although these are not the same, as was discussed above. For example, intestinal permeability changes do not necessarily reflect changes in mucus secretion, antimicrobial production, or IgA secretion. In this section we discuss the use of markers in blood, feces, or urine that could be used to assess intestinal permeability in animals and humans (Table 2).

**Table 2.** Methods for assessment of intestinal permeability, epithelial integrity, and mucus properties

Method	Test Molecules	Applicable Sites	Biological Sample	Comments
*Methods of assessing intestinal permeability*				
Measurement of short-circuit current in Ussing chambers	Ion transport	Whole intestine	Biopsies	Invasive, fresh tissue or biopsy material needed, duration of experiment limited to 2 h
Dual sugar quantification using mass spectrometry	Oligosaccharides of different MW (e.g., lactulose/ mannitol)	Small intestine	Urine	Time consuming, affected by GI motility, renal function
Quantification using mass spectrometry	PEGs, 4,000/400 kDa	Whole intestine	Urine	Equivalent performance to dual sugar test reported, time-consuming
^51^Cr-EDTA radioisotope activity	^51^Cr-EDTA	Whole intestine	Urine	Radioactivity
LAL assay	Endotoxin (LPS)	Whole intestine	Plasma	Standardization difficult in human samples
*Methods of assessing epithelial integrity and intestinal inflammation*				
Mass spectrometry	Citrulline, an epithelial amino acid not incorporated into protein	Small intestine	Plasma	Validated as a useful indicator of loss of small bowel epithelial cell mass in transplant recipients and chemotherapy; not likely to be sensitive enough for healthy subjects
ELISA	I-FABP	Jejunum	Plasma	Studies mostly in patients with small intestinal inflammation
ELISA	I-BABP	Ileum	Plasma	Studies mostly in patients with small intestinal inflammation
ELISA	L-FABP	Whole intestine	Plasma	Expressed in kidney and liver
ELISA	Zonulin, claudin 3 (potentially other tight junction proteins)	Whole intestine	Plasma	Few studies
Confocal fluorescence microscopy of TJ proteins	TJ proteins	Whole intestine	Biopsy or surgical tissue	Requires confocal microscopy and specialized image analysis methods; labor intensive
ELISA	Calprotectin	Whole intestine	Feces	Released by activated neutrophils at inflamed sites; evaluated in colitis studies
ELISA	LCN-2	Whole intestine	Feces	Expression of LCN-2 upregulated in epithelial cells by inflammation; also expressed in neutrophils
Quantification by real-time PCR	miRNAs upregulated in inflamed enterocytes	Whole intestine	Feces or plasma	Potential new markers but few studies and mainly in cancer patients
Morphological studies using paraffin fixed tissue and H&E staining	Tissue appearance and morphology	Whole intestine	Biopsy or surgical tissue	Invasive, used to assess severity of mucosal damage in patients
*Methods of assessing mucus thickness and penetrability*				
Fluorescent microscopy of mounted tissue ex vivo	Permeability of fluorescent beads through mucus	Whole intestine	Biopsy or surgical tissue	Fresh tissue required, specialized microscopy set up required
Carnoy fixation and mucus detection using PAS/Alcian blue or antibodies	Secreted mucus,	Whole intestine	Tissue sample	Invasive but can be used to measure mucus thickness and quantify goblet cell numbers, can be combined with FISH staining of microorganisms; human biopsy sampling method may not preserve mucus layer

51 Cr-EDTA, chromium-labeled EDTA; FABP, fatty acid binding protein; FISH, fluorescent in situ hybridization; GI, gastrointestinal; H&E, hematoxylin and eosin; I-BABP, ileal bile acid-binding protein; I-FABP, intestinal fatty acid-binding protein; LAL, limulus amebocyte lysate assay; LCN-2, lipocalin-2; L-FABP, liver-type fatty acid-binding protein; LPS, lipopolysaccharide; miRNA, microRNA; MW, molecular weight; PAS, period acid Schiff; PEG, polyethylene glycol; TJ, tight junction.

Permeability of the small intestine is commonly evaluated by measurement of intestinal permeation and urinary excretion of orally administered water-soluble, nonmetabolizable sugars that differ in size. Typically, these assays use oligosaccharides of a large size [e.g., lactulose or polyethylene glycols (PEGs) of 1,500 to 4,000 kDa] and low-molecular-weight sugars such as mannitol and L-rhamnose, or low-molecular-weight PEG (400 kDa) (Table 2). The larger sugar molecules such as lactulose are assumed to permeate paracellularly when the intestinal barrier is compromised, whereas the smaller molecules such as mannitol are assumed to permeate both transcellularly and paracellularly so that the ratio of these two sugars in plasma or excreted in the urine reflects intestinal permeation, taking into account differences in the surface area of the epithelium. Because the sugars used in these permeability assays can be metabolized by colonic bacteria, their excretion in the urine is assumed to predominantly reflect permeation of the small intestine (175). Sucralose has been used instead of lactulose as a measure of whole gut permeability (67). The influence of preabsorptive factors such as small-bowel transit time was shown not to influence the outcome of the dual-sugar permeability test. Recently, renal clearance of rhamnose but not lactulose was shown not to depend on the quantity of these sugars in the circulation (67). Thus, a relative increase in permeation of lactulose and rhamnose in the small intestine may be underestimated in this test (267a).

In a review of the clinical applications of the dual-sugar permeability test it was reported to be useful for screening of small intestinal disease, prognosis, and response to treatment, especially in celiac disease (256). However, it was not recommended as a predictor of nonsteroidal anti-inflammatory drug-related upper gastrointestinal damage or as a marker of disease activity in IBD (256). The dual-sugar permeability test has been used to measure increased intestinal permeability in a human cohort before the onset of type 1 diabetes (29), suggesting the method may also be useful to assess “gut health” in nutritional intervention studies involving healthy subjects. In the diabetes study mentioned above (29), at-risk individuals having B-cell autoantibodies showed an increased lactulose-to-mannitol ratio, which is indicative of increased paracellular permeability. Interestingly, this finding is supported by a further study (227), which showed that serum zonulin concentrations correlated with increased intestinal permeability in vivo in patients with type 1 diabetes. Moreover, serum zonulin levels were even increased in B-cell autoantibody-positive individuals at risk for type 1 diabetes (227). This indicates that serum levels of TJ proteins might also be promising biomarkers of epithelial integrity. An alternative method for measuring intestinal permeability is the ^51^Cr-ETA test, which is performed by calculating the percentage of recovery from urine of an oral dose of ^51^Cr-EDTA (Table 2). It has been used to detect increased intestinal permeability in Crohn’s disease, celiac disease, and nonalcoholic fatty liver disease (188). Urinary excretion of PEGs has also been used to study intestinal permeability changes in patients with alcoholic liver disease and Crohn’s disease compared with healthy controls (71, 118). A recent study using PEG and lactulose/rhamnose sugar probes to measure intestinal permeability in healthy human volunteers given indomethacin showed that both methods give comparable results in a clinical setting (269).

Potential biomarkers of epithelial integrity that have a causal relationship with permeability and innate barrier functions would be molecules produced by epithelial cells. One example is the fatty acid-binding proteins (FABPs), which are small cytosolic proteins found in enterocytes of both the small and large intestines. Three different types of FABPs are found in the intestine (Table 2). Intestinal-FABP (I-FABP) is found mainly in the enterocytes of the jejunum and in low amounts in the colon. Liver-type FABP is found throughout the intestine but also in the liver and kidney. The ileal bile acid-binding protein (I-BABP) is found only in the ileum. All protein markers can be measured in plasma and urine using ELISA. To date they have been used only in clinical studies; for example, in patients with celiac disease, intestinal ischemia, necrotizing colitis (3, 211, 212, 253, 273), and in patients who have undergone liver transplant in which their prognostic value was demonstrated (182). Alternative small-molecule markers of intestinal epithelial integrity include TJ proteins. Studies in patients with Crohn’s disease have shown a relationship between levels of claudin-3 in the urine of patients with IBD or necrotizing colitis (90, 296) (Table 2). In type 1 diabetes, impairment of the gut barrier is considered one of the factors contributing to the onset of the disease, and increased serum zonulin precedes the onset of the disease (227). Similarly, significantly greater amounts of zonulin were measured in the serum of patients with celiac disease and obesity than in healthy controls (68) (Table 2).

Plasma levels of citrulline, an amino acid produced by small intestinal enterocytes from glutamine but not incorporated into proteins, is considered a useful marker of functional enterocyte mass (Table 2). It has been validated as a useful marker of small bowel epithelial cell mass in hemopoietic stem cell transplant recipients suffering from severe oral and gastrointestinal mucositis following intensive myeloablative therapy (53). More recently, citrulline was established as a valuable marker for chemotherapy-induced mucosal barrier injury in pediatric patients (268). The sensitivity and specificity of citrulline as a marker of were better than the sugar permeability tests (163).

Detection of the inflammatory marker calprotectin in feces has also been used as a surrogate marker of epithelial integrity in many disease studies because excessive intestinal inflammation is known to increase epithelial permeability. Calprotectin is highly expressed in neutrophils and macrophages, and the increased permeability of an inflammed mucosa allows calprotectin released from activated neutrophils to be released into the intestinal lumen (49, 50). A sensitive noninvasive marker of intestinal inflammation is lipocalin 2 (LCN2) (43) (Table 2), which binds to bacterial siderphore enterochelin, thereby limiting the growth of bacteria in the iron-limited environment of the gut (75). Elevated amounts of LCN2 have been detected in mice fed a high-fat, high-salt diet and in colitis models (5). Recent studies of patients with IBD showed that LCN2 was among the 10 most upregulated genes in both active ulcerative colitis and active Crohn's disease compared with healthy controls (192). LCN2 protein was found in both epithelial cells and infiltrating neutrophils, whereas mRNA synthesis was detected only in epithelial cells. Thus, fecal LCN2 is another interesting putative biomarker for intestinal inflammation.

Recently, noncoding microRNAs (miRNAs) such as miRNA-222, miRNA-30, miRNA-29b, miRNA-503, miRNA-195, and miRNA-320a have been demonstrated to play a role in the regulation of epithelial regeneration, protection, and epithelial barrier function (194). The mechanisms through which these miRNAs modulate the stability and translation of target mRNAs are still being unraveled, but they have future potential to be used as fecal biomarkers of intestinal function and diseases such as IBD and cancer (125, 195).

## Ex Vivo and Histological Approaches to Studying Intestinal Permeability or Gut Barrier Properties

The Ussing chamber allows animal or human intestinal tissue to be mounted such that the apical side is isolated from the basolateral side, and by filling each compartment with Ringer solution the short-circuit current can be used as an indicator of ion transport across the epithelium [reviewed by Herrmann and Turner (103)]. The technique allows the permeability of tissue from biopsies of patients and healthy subjects to be measured. The influence of nutrients and other factors on intestinal ion permeability can also be studied. A drawback is that fresh tissue is needed and must be used immediately. Experiments are typically limited to about 2 h after mounting to avoid artifacts from necrosis of the tissue.

Histological examination using biopsies or resected tissue from animals and humans is also a common experimental way of studying aspects of barrier function (103, 162). For example, immunofluorescent antibody detection of TJs or adherens junctions has been used to assess altered barrier dysfunction in disease states (26, 288) (Table 2).

Carnoy fixation and paraffin embedding of intestinal tissue followed by immunofluorescent antibody or periodic acid Schiff/Alcian blue staining of MUC2 can be used to assess mucus thickness in the colon of small rodents, but the technique is dependent on the presence of a fecal pellet in the intestine, otherwise the mucus is easily displaced during the fixation and staining procedure (Fig. 2). Detection of bacteria in the same sections with fluorescence in situ hybridization probes to conserved or specific 16S RNA gene sequences also enables the location of microbiota to be localized in the intestine (94, 119, 120). Ex vivo techniques have also been developed for investigating mucus permeability using mounted tissue explants and a specialized fluorescent microscopy setup to visualize the spatial distance of fluorescence beads from the epithelium (17, 65) (Table 2).

## Main Conclusions

The gut barrier plays a crucial role by spatially compartmentalizing bacteria to the lumen. This is achieved through the production of a secreted mucus that limits penetration of the bacteria and is fortified by the production of antimicrobial peptides and proteins that kill or inhibit growth of bacteria in proximity to the epithelium. Secretory IgA is abundantly produced in the gut and contributes to the exclusion of bacteria from the epithelial surface primarily through agglutination. With the exception of sIgA, expression of these protective barrier factors is largely controlled by innate signaling mechanisms involving pattern-recognition receptor signaling in response to binding of conserved microbial molecular ligands. Overreaction is regulated by inherent feedback mechanisms and the controlled expression of pattern recognition receptors in the epithelium. Additionally, the mucosa maintains a distinct noninflammatory tone through the steady-state induction of mucosal regulatory T cells and tolerogenic dendritic cells in LP and mucosal lymphoid tissues. Collectively, these mechanisms contribute to the homeostasis of gut barrier function and mucosal immunity (218).

Antibiotic treatment alters microbiota composition profoundly leading to diminished goblet cell function, a reduction of the inner mucus layer, and loss of antimicrobial peptides and immune tolerance [for a detailed review see (290)], which may be due in part to the reduced stimulation of the innate immune system. Antibiotic treatment or other environmental factors may also promote greater numbers of pathobionts that have the capacity to cause harm in compromised or genetically susceptible individuals. Over the past 5 yr, several prominent commensal-host interactions have been characterized in vivo and in vitro. On the basis of this research it is becoming clear that certain bacterial species can either promote or attenuate inflammatory responses (112, 180, 244). In a healthy animal or person these opposing host interactions are probably balanced, but in several intestinal disorders, host-microbe interactions may be skewed in a direction that contributes to pathophysiological processes (44).

The paracellular permeability of the intestine to ions and small molecules is dependent on the intestinal location and is controlled by the TJ protein complexes that connect adjacent cells in the epithelium. Inflammatory stimuli can increase permeability of the epithelium through contractions of the perijunctional actomyosin ring that is connected to the TJ complex or via altered TJ protein composition or dynamics. Chronic changes in epithelial permeability are considered to contribute to the pathophysiology of several intestinal disorders by allowing antigens or inflammatory stimuli to enter the LP and perturb homeostasis. In obesity, for example, altered epithelial permeability and permeation of the gut by LPS can lead to increased levels in the plasma and insulin-resistant states (117).

The proper functioning of the gut barrier has implications beyond the gut and mucosal immunity affecting metabolism, adaptive immunity, the enteric nervous system, and the brain. The intestinal barrier function plays a central role in how gut-brain interaction affects the immune system. Deterioration of the intestinal barrier function may lead to increased and prolonged mucosal immune activation and, consequently, to increased afferent sensory signaling and abdominal complaints. Brain function affects the intestinal barrier partly by activation of the HPA-axis and both mast cell-dependent as well as mast cell-independent mechanisms. Furthermore, the role of microbial metabolites such as butyrate have gained interest in this respect.

Several biomarkers have been developed to measure intestinal permeability and epithelial integrity in blood, feces, and urine. The dual-sugar permeability assay is the most widely used method of assessing permeability. The method is considered to mainly reflect small intestinal permeability, and validated assays for colonic barrier function remain to be fully developed. PEGs of 1,500 to 4,000 kDa are alternatives to the sugar probes and have shown comparable results in a clinical setting (269). Biomarkers that can be measured in feces include inflammation-induced proteins LCN2 and calprotectin, but to date, they have been used only to study epithelial integrity in patients.

Several plasma and urine biomarkers of epithelial integrity have been evaluated in patients, including the intestinal FABPs and TJ proteins, which can be measured in plasma and urine. I-FABP is found primarily in enterocytes of the small intestine, and I-BABP is found only in the ileum, offering the possibility of measuring integrity at specific intestinal locations. Citrulline, an amino acid produced by epithelial cells, has been validated as a useful marker of loss of enterocyte mass in severe conditions of intestinal damage. However, the biomarker is not sufficiently sensitive to assess the extent of intestinal damage and is likely to be of limited use in nonclinical situations.

Although some of the markers reviewed above may be useful for determining prognosis in treatment of diseases, few examples exist of their use in assessing the effects of nutrition in healthy subjects. One exception is the application of the dual-sugar and PEG permeability markers in healthy volunteers given indomethacin, which temporally increases small intestinal permeability (269). Challenge studies of this kind might therefore be useful in assessing the effects of nutrition on intestinal permeability. Regardless of the marker used, there will be a normal range of values defining the “bandwidth” of health in the general population that is due to differences in genetic make-up and environmental and lifestyle factors. Values close to or outside the normal boundaries may modify disease risk or contribute to pathological processes. Therefore, including these potential biomarkers in future prospective cohort studies involving healthy subjects, will help to establish normal ranges of biomarker measurements, their variability within a subject over time, and their predictive values for onset of diseases related to gut barrier dysfunction. Ultimately, such markers and assays could be used to assess associations between particular nutritional traits and gut barrier function or experimentally to assess the effect of a specific nutritional intervention. Thus, validating markers to assess intestinal health in the general population is clearly an important goal for the future.

## GRANTS

This work was conducted by an expert group of the European branch of the International Life Sciences Institute, ILSI Europe. This publication was coordinated by the Probiotics Task Force. The research reported is the result of a scientific evaluation in line with ILSI Europe's framework to provide a precompetitive setting for public-private partnership (PPP). ILSI Europe facilitated scientific meetings and coordinated the overall project management and administrative tasks relating to the completion of this work. For further information about ILSI Europe, please email info@ilsieurope.be or call +32 2 771 00 14. The opinions expressed herein and the conclusions of this publication are those of the authors and do not necessarily represent the views of ILSI Europe nor those of its member companies.

## DISCLOSURES

M.D. is an employee of Danone, A.M. and C.L.G.R. are employees of Nestlé, A.N. is an employee of FrieslandCampina, and A.M. was an employee of ILSI Europe. The remaining authors have no conflicts of interest.

## AUTHOR CONTRIBUTIONS

J.M.W. and R.J.B. prepared figures; J.M.W., R.J.B., M.D., T.T.M., F.T., P.D.C., V.T., J.D., W.M.d.V., A. Mercenier, A.N., A. Méheust, and C.L.G.-R. drafted manuscript; all authors edited, revised, and approved the final manuscript.
